# Targeting KRAS Sensitizes Ferroptosis by Coordinately Regulating the TCA Cycle and Nrf2‐SLC7A11‐GPX4 Signaling in Hepatocellular Carcinoma

**DOI:** 10.1002/smmd.70005

**Published:** 2025-05-06

**Authors:** Jiaxin Zhang, Zuojia Liu, Wenjing Zhao, Chang Li, Fei Liu, Jin Wang

**Affiliations:** ^1^ State Key Laboratory of Electroanalytical Chemistry Chinese Academy of Sciences Changchun Institute of Applied Chemistry Changchun China; ^2^ School of Chinese Medicine Hong Kong Traditional Chinese Medicine Phenome Research Center Hong Kong Baptist University Hong Kong China; ^3^ Hepatobiliary Hospital of Jilin Province Changchun China; ^4^ Center for Theoretical Interdisciplinary Sciences Wenzhou Institute University of Chinese Academy of Sciences Wenzhou China; ^5^ Department of Chemistry and Physics Stony Brook University Stony Brook New York USA

**Keywords:** ferroptosis, hepatocellular carcinoma, KRAS, metabolomics, NSC48160

## Abstract

Oncogenic KRAS, a notorious driver of cancer progression, remains a therapeutic challenge. In hepatocellular carcinoma (HCC), KRAS overexpression correlates with tumor aggressiveness. Here, we demonstrate that NSC48160 induces HCC cell death by suppressing KRAS expression. Metabolomic profiling revealed that NSC48160 significantly enhances intracellular tricarboxylic acid (TCA) cycle activity and fructose metabolism, disrupting redox homeostasis, and triggering ferroptosis. Combining NSC48160 with the SLC7A11 inhibitor HG106 synergistically eliminated HCC cells in vitro and suppressed tumor growth in vivo. Mechanistically, NSC48160 indirectly inhibits the Nrf2‐SLC7A11‐GPX4 axis, as evidenced by ferroptosis‐pathway array assays. Specifically, NSC48160 downregulates Nrf2 expression, thereby suppressing its downstream targets GPX4 and SLC7A11, ultimately promoting ferroptosis. Our findings establish NSC48160 as a novel KRAS inhibitor that induces ferroptosis through metabolic and redox reprogramming, offering a promising therapeutic strategy for KRAS‐driven HCC.

**Summary**

- Pharmacological agent NSC48160 inhibits the development of HCC by promoting the TCA cycle, elevating the OCR, and an augmented generation of ROS.
- NSC48160 impedes the proliferation of HCC cells via KRAS‐mediated ferroptosis, linked with the regulatory triad of the Nrf2‐SLC7A11‐GPX4 signaling axis.
- NSC48160 modulates tumor metabolism and the accompanying shifts in redox balance, which are posited as a promising therapeutic avenue.

**Abbreviations**
CETSA: cellular thermal shift assay
ECAR: extracellular acidification rate
GPX4: glutathione peroxidase 4
GSH: glutathione
HCC: hepatocellular carcinoma
KRAS: Kirsten rats arcomaviral oncogene homolog
Nrf2: nuclear factor erythroid 2‐related factor 2
OCR: oxygen consumption rate
ROS: reactive oxygen species
SLC7A11: solute carrier family 7 member 11T
TCA: tricarboxylic acid cycle

## 1 Introduction

Hepatocellular carcinoma (HCC), the most common primary liver malignancy, ranks as the sixth most prevalent cancer and third leading cause of cancer‐related mortality worldwide [1, 2]. Despite therapeutic advancements, the 5‐year survival rate remains dismal due to tumor heterogeneity and therapy resistance [3, 4, 5]. The inherent plasticity and genetic instability of neoplasms confer resistance to all forms of monotherapy. This underscores the imperative need for the discovery of novel drugs and the identification of potential therapeutic targets based on the pathogenesis of HCC. The susceptibility of cells to ferroptosis is intricately associated with various cellular metabolic pathways, particularly those governing the metabolism of amino acids, iron, and polyunsaturated fatty acids. Additionally, the biosynthetic pathways for critical components such as glutathione, phospholipids, and NADPH play a significant role in modulating ferroptosis. Metabolic reprogramming, particularly enhanced glucose and glutamine utilization is a hallmark of HCC progression. KRAS, a key oncogenic driver, orchestrates this metabolic shift by promoting TCA cycle flux and glutaminolysis to sustain redox homeostasis. The oncogene KRAS emerges as a critical regulator in orchestrating this metabolic reprogramming. The regulation of glutamine metabolism by KRAS has significant implications for the TCA cycle, a pathway that is essential for nucleotide biosynthesis, thereby supporting cellular growth and survival. Tumors driven by KRAS exhibit a critical dependence on glutaminase to maintain redox homeostasis [6]. KRAS‐mutant HCC cells exhibit upregulated Nrf2, a master regulator of ferroptosis resistance through many ferroptosis related metabolic processes, such as SLC7A11‐mediated cystine uptake and GPX4‐dependent lipid peroxide detoxification. Targeting KRAS‐induced metabolic vulnerabilities may therefore provide a therapeutic breakthrough.

The proliferation capacity of KRAS‐mutant hepatocellular carcinoma cells is notably enhanced [7, 8]. During rapid growth, these cells experience oxidative stress, which subsequently leads to the upregulation of the transcription factor Nrf2. Nrf2 has been identified as a key regulator of ferroptosis due to its ability to target a broad array of genes involved in the ferroptosis signaling cascade [9]. In the metabolism of amino acids, this modulation is achieved through inhibiting solute carrier family 7 member 11 (SLC7A11) [10], precipitating a state of cysteine scarcity and, consequently, a deficiency in substrate synthesis for glutathione (GSH), thereby impairing the functional efficacy of the antioxidant enzyme GPX4 [11, 12]. In instances where lipid peroxides are unable to undergo metabolic breakdown via the GPX4‐catalyzed glutathione reductase reaction, divalent iron ions act as catalysts for lipid oxidation, culminating in the production of ROS. This biochemical cascade significantly fuels the initiation of ferroptosis [13, 14]. Suppressing KRAS expression has been demonstrated to instigate the activation of the ferroptosis pathway within metabolic processes [15, 16].

In the present study, we conducted a comprehensive metabolomics analysis to explore the metabolic networks within tumor tissues, with the goal of identifying metabolic vulnerabilities that could be exploited for the therapeutic intervention of HCC. Applying a targeted chemical screening process, a small molecule NSC48160 was identified as an efficacious inhibitor of KRAS target, which regulated ROS production, mitochondrial stress, and the induction of growth arrest ultimately, specifically within HCC cells under the upregulation of the TCA cycle. This study elucidates that pharmacological agent NSC48160 can precipitate ferroptosis and redox balance via modifications in their metabolic landscape, thereby impeding the proliferation of HCC cells via KRAS‐mediated ferroptosis, which is intricately linked with the regulatory triad of the Nrf2‐SLC7A11‐GPX4 signaling axis, employing both in vitro and in vivo methodologies, thus making it a potential therapeutic drug.

## 2 Materials and Methods

### 2.1 Materials and Reagents

NSC48160 was supplied by the NCI/DTP Open Chemical Repository (http://dtp.cancer.gov). Before use, 10 mg/mL NSC48160 in phosphate‐buffered saline (PBS) was diluted to appropriate concentrations with a cell culture medium. DMEM culture medium and fetal bovine serum (FBS) were purchased from Gibco (Grand Island, NY). Specific siRNAs were purchased from Ribo‐Bio (Guangzhou, China). The other reagents can be seen in supporting information (Table 1).

**TABLE 1:** Components of materials and reagents.

Materials and reagents		
Materials	Source	Identifier
Small molecule compound NSC48160	NCI/DTP open chemical repository	http://dtp.cancer.gov
Phosphate‐buffered saline (PBS)	Beyotime (Shanghai, China)	C0221A
DMEM culture medium	Gibco (Carlsbad, CA, USA)	11965092
RPMI 1640 medium	Gibco (Carlsbad, CA, USA)	12633012
Fetal bovine serum (FBS)	Biological industries (Kibbutz Beit Haemek, Israel)	04‐001‐1A
Penicillin‐streptomycin	Gibco (Carlsbad, CA, USA)	15140122
Nrf2 antibody	Cell signaling technology	#12721
KRAS antibody	Cell signaling technology	#71835
SLC7A11 antibody	Abcam (Boston, MA)	ab307601
GPX4 antibody	Abmart (Shanghai, China)	T56959
β‐Actin antibody	Cell signaling technology	#4970
Horseradish peroxidase (HRP)‐conjugated goat anti‐rabbit secondary antibodies	ZSGB‐BIO (Beijing, China)	ZB‐2306
Polyvinylidenedifluorid (PVDF) membranes	Millipore (Billerica, MA, USA)	ISEQ00010
TWEEN 20	Sigma‐Aldrich Corp. (St. Louis, MO, USA)	P9416
TBS (10X, premixed powder)	Beyotime (Shanghai, China)	ST667‐1L
riboFECT CP transfection reagent	RiboBio (Guangzhou, China)	C10511‐05
Cell counting kit‐8	Beyotime (Shanghai, China)	C0038

### 2.2 Cell Culture

Human hepatoma‐derived cell lines HepG2, SMMC‐7721, and BEL‐7402 were obtained from the Shanghai Type Culture Collection. These cell lines were regularly tested for *mycoplasma* contamination. To maintain these cell lines, DMEM/1640 media supplemented with 10% FBS was used and incubated at 37°C in a 5% CO_2_ incubator.

### 2.3 Western Blots (WB) Analysis

Equal amounts of total cellular proteins were separated by SDS‐PAGE and transferred to polyvinylidenedifluorid (PVDF) membranes (Millipore, MA). Thereafter, membranes were blocked in 5% non‐fat dry milk in TBST containing 0.1% Tween‐20 for 1 h at room temperature (RT). Subsequently, membranes were probed with the relevant primary antibodies (1:1000 dilution) overnight at 4°C. After washing, they were exposed to the appropriate HRP‐conjugated secondary antibodies (1:1000 dilution) for 1 h at RT. *β*‐Actin was loaded as an internal control. Immunoreactive complexes were visualized using a Chemiluminescence detector (DNR, KiryatAnavim, Israel). Density scanning of each protein band was performed using Image Lab software (Bio‐Rad, Hercules, CA).

### 2.4 siRNA Transfection in Cells

The suppression of SLC7A11 activity was achieved through RNA interference methodologies. Target sequences for siRNA are as follows: siSLC7A11, GGAAGAGATTCAAGTATTA and siSLC7A11‐1, GCTGCTGCTTTCAAATGCA. They were transfected by riboFECT CP transfection reagent (Guangzhou, China) according to the manufacturer's instructions. Cells transfected with a controlled nonspecific siRNA were used as control for direct comparison. The cells were inoculated with 1 × 10^5^ cells for 24 h before transfection, the cell fusion was 70%, the final concentration of siRNA transfected cells was 100 nM, the cells were incubated at room temperature for 15 min, and the cell plates were incubated at 37°C in a 5% CO_2_ incubator for 48 h. After transfection, the expression level of SLC7A11 was quantitatively analyzed via western blot analysis.

### 2.5 Real‐Time Cell Analysis (RTCA) Assay

The proliferation of cells was analyzed in real‐time using RTCA. To elaborate, we seeded 10^4^ cells per well in 16‐well E‐plates from Acea Biosciences and added either a control medium or medium supplemented with NSC48160. The xCELLigence Real‐Time Cell analyzer monitored the plates for 80 h. The xCELLigence system automatically monitored the impedance value of each well and expressed it as a cell index value.

### 2.6 Measurement of OCR and ECAR in HepG2 Cells

Real‐time measurements of oxygen consumption rate (OCR) and extracellular acidification rate (ECAR) were performed with an XFp Extracellular Flux analyzer (Agilent Seahorse Bioscience, MA). OCR and ECAR values were measured using the Cell Mito Stress Test Kit (Agilent, 103010‐100) and Glycolysis Stress Test Kit (Agilent, 103017‐100), respectively. All OCR and ECAR values were normalized to the protein content of each well.

### 2.7 Cell Viability and Proliferation Assay

Cells were seeded at a density of 5 × 10^3^ cells/well into 96‐well plates and treated with the indicated NSC48160 concentrations for 24 h, respectively. Therefore, 10 μL/well of Counting Kit‐8 (CCK‐8) reagent was added, and cells were incubated at RT for 1 h. The absorbance was analyzed on a microplate reader (Tecan, Hombrechtikon, Switzerland) at 450 nm wavelength.

### 2.8 Tumor Xenograft Models

Twenty female athymic nude mice, aged 6 weeks and weighing 20 ± 1.0 g, were obtained from Beijing Vital River Laboratory Animal Technology Co. Ltd (Beijing, China) and placed in sterile micro isolator cages for 1 week of acclimation. SSMMC‐7721 cells (2 × 10^6^), along with SMMC‐7721 cells with knockdown‐KRAS, were implanted subcutaneously into the mice using FBS medium, and the tumors were allowed to grow to 80 mm^3^. The mice were randomized into four groups, with five mice in each group, and drug treatment began at this point. Over a 7‐day period, 0.9% saline 100 μL containing NSC48160 (50 mg/kg) was administered every 2 days. The control group received an equivalent volume of saline solutions. For inducible subcutaneous xenograft models, tumor volume was recorded by caliper measurements using the formula (long diameter [mm]) × (short diameter [mm]) 2 × (1/2). At the end of the experimental period, mice were anesthetized, and all mice were sacrificed. After successfully establishing the xenograft models of nude mice, the survival mice were recorded every day. The tumor growth curve of the nude mice was plotted with time (day) as the abscissa and tumor volume as the ordinate. Tumors were dissected and weighed. All experimental procedures were approved by the Animal Care and Use Committee of Wish Detection Technology Co. Ltd, Changchun, China, and performed in strict accordance with Legislation Regarding the Use and Care of Laboratory Animals in China.

### 2.9 Immunohistochemical Analysis

Mice were sacrificed and tumors were excised. 4‐μM‐thick tumor sections fixed of paraffin‐embedded were deparaffinized in xylene and rehydrated in graded alcohol. Tumor sections were then incubated by primary antibodies (KRAS, Nrf2, SLC7A11, and GPX4) overnight at 4°C with gentle shaking. After washing with PBST, tumor sections were incubated with the secondary antibody for 1 h at RT. After washing with PBST, sections were exposed to diaminobenzidine (Abcam). Images were obtained by an inverted phase microscope (Olympus X71, Hessen, Germany).

### 2.10 Surface Plasmon Resonance (SPR)

Reichert SR7500DC SPR system instructions were followed for the experiment (Reichert, New York, USA). Before immobilization on a sensor chip, KRAS protein was diluted to a final concentration (100 μM) in sodium acetate. It was also injected through the channel. Then, 1M pH 8.5 ethanolamine was injected over channels to cap the chip's surface. NSC48160 binding affinity with NSC48160 was determined by injecting different doses of NSC48160 into KRAS immobilized chambers (1–128 μM). Results were analyzed by TraceDrawer software (Reichert, New York, USA).

### 2.11 Molecular Docking Analysis

We obtained crystal data for HRASG60A (PDB ID: 1XCM) and amino acid sequences for carbohydrate‐binding modules from the RCSB Protein Data Bank. To visualize and process these proteins, we utilized Pymol V2.5.2 software. The protein backbone is depicted in cartoon form with each subunit colored differently.

### 2.12 Cellular Thermal Shift Assay (CETSA)

Binding between NSC48160 and KRAS was confirmed through CETSA analysis. HCC cells were exposed to DMSO and NSC48160 (36 μM) for 24 h before being carefully rinsed with protease inhibitor‐containing PBS solutions. Afterward, the samples were heated for 3 min at various temperatures (ranging from 47°C to 63°C) using a heat block. Finally, the extracted proteins were analyzed through Western blotting.

### 2.13 Metabolomics Analysis

The total samples were extracted from the tumor in the control and NSC48160 groups (*n* = 6). Each sample consisted of 200 ± 10 mg of tumor tissue, which was homogenized in a 1 mL solution containing methanol and an internal standard mixture. The resulting samples were then analyzed using liquid chromatography‐tandem mass spectrometry (LC‐MS/MS) with a QTRAP System manufactured by SCIEX in Framingham, USA. The metabolomics analysis was carried out by Metware Technology in Wuhan, China.

### 2.14 Ferroptosis Assay

Ferroptosis‐pathway Array was performed to explore the signaling pathways that were regulated by NSC48160 in HCC cells. The assay was conducted according to the manufacturer's protocol. Experiments were repeated three times, and the data are presented as the mean ± SD.

### 2.15 Mitochondrial Morphology

To obtain cellular specimens for transmission electron microscopy (TEM) analysis, cells were isolated through centrifugation, with the pellet size approximately equivalent to half the size of a typical green bean. After isolation, the cellular samples were fixed using a 2.5% glutaraldehyde solution for a minimum duration of 6 hours to ensure adequate preservation of cellular architecture. For the purpose of visualizing the internal structure of the cells under TEM, copper mesh supports were prepared and utilized to hold the specimens.

### 2.16 Statistical Analysis

Data were analyzed using SPSS software (IBM SPSS Statistics, USA). Statistical analyses were performed using the Student's t‐test. All values are presented as the mean ± standard error of the mean (SEM). *p < 0.05 and **p < 0.01 were considered statistically significant.

## 3 Results

### 3.1 KRAS Is Involved in the Progression of HCC

**FIGURE 1:**
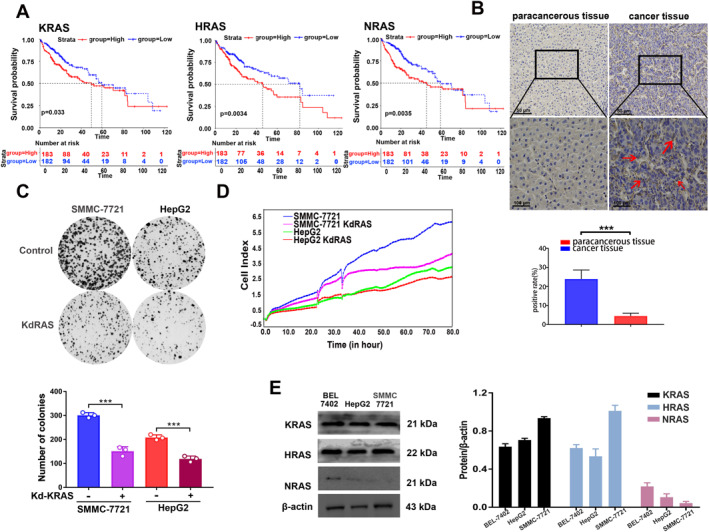
The effects of KRAS, HRAS, and NRAS in HCC. (A) A Kaplan–Meier survival curve for overall survival in liver cancer patients according to the tumor expression of KRAS, HRAS, and NRAS. (B) Immunohistochemical analyses of KRAS were examined in paracancerous tissues and cancer tissues. Quantitative analyses were examined. Scale bar: 50 μm (up) or 100 μm (down). *n* = 3. (C) The SMMC‐7721 and HepG2 cells were analyzed under KRAS knockdown for 7 days. Clone formation and quantitative analyses were examined. *n* = 3. (D) Dynamic proliferation profiling was detected in SMMC‐7721 and HepG2 cells treated with KRAS knockdown over 80 h using the xCELLigence system. (E) The qualitative and quantitative expression of KRAS, HRAS, and NRAS were examined in SMMC‐7721, BEL‐7402, and HepG2 cells. *p < 0.05, **p < 0.01, ***p < 0.001.

To elucidate the relationship between oncogenic RAS and HCC progression and the potential utility of the subtyping signatures, we compared prognosis among three clinical subsets, including KRAS, NRAS, and HRAS. The clinical data downloaded from the TCGA (The Cancer Genome Atlas) transcriptome datasets include 50 normal lives and 371 HCC samples. The survival of patients with three subsets was significantly different (Kaplan‐Meier analysis, log‐rank *p* < 0.05). Low expression of KRAS, HRAS, or NRAS showed a better outcome compared to the high expression of the three genes (Figure 1A, Supporting Information S1: Figure S1). Especially, the expression of KRAS is significantly correlated with the prognosis of disease, owning a profound influence on patient survival outcomes. The enhanced expression of KRAS was found in tumor tissues from human subjects with HCC compared to cancer‐adjacent normal tissues, showing that the KRAS and its downstream pathways are also highly activated in HCC (Figure 1B). To confirm whether the human HCC cells recapitulated the characteristics of KRAS activities consistent with human HCC tumors, a KRAS‐knockdown assay was performed in HCC cell lines HepG2 and SMMC‐7721. Apparently, KRAS‐knockdown hampered cell viability consistently (Figure 1C) and delayed cell growth as determined by the real‐time xCELLigence system remarkably (Figure 1D). Notably, the expression of KRAS proteins had a much higher level in the HepG2 and SMMC‐7721 (HCC cells), while the other two HRAS and NRAS proteins were at lower levels (Figure 1E). Collectively, these results demonstrated that KRAS was prominently expressed and essentially indispensable in HCC.

### 3.2 Identification of NSC48160 as an Effective KRAS Inhibitor

**FIGURE 2:**
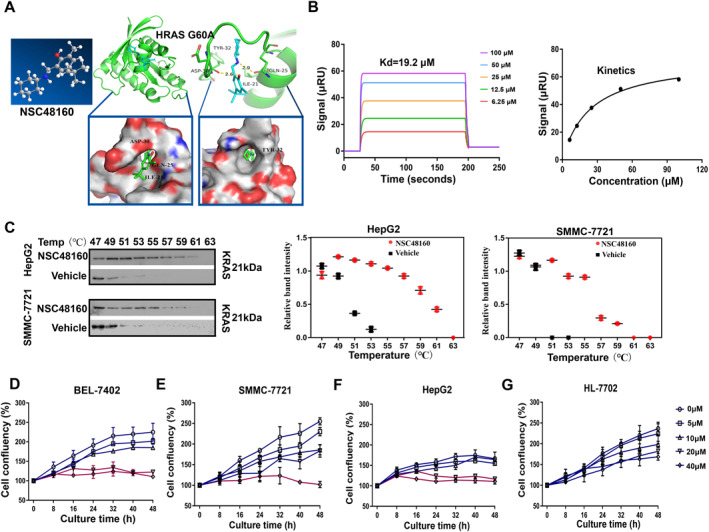
NSC48160 induces cell death by targeting KRAS. (A) Structure models of HRASG60A (PDB ID: 1XCM) and superposition of the two structures as ribbon (top) and surface (bottom) representations. (B) The interaction between NSC48160 and HRASG60A was analyzed using a surface plasmon resonance (SPR) assay. A graph of equilibrium RIU responses versus compound concentrations was plotted (right). (C) CETSA was used to evaluate the binding between NSC48160 and KRAS at the thermodynamic level. The expression of KRAS was detected by western blotting. (D–G) Cell growth curves of BEL‐7402, SMMC7721, HepG2 cells, and HL7702 cells for 48 h were treated with different doses of NSC48160; *n* = 3, performed in triplicate.

Considering the functions of KRAS in tumors, developing KRAS‐targeted agents can enhance responsiveness to adjuvant chemotherapy of HCC [17]. Improvements in drug design have eventually led to the development of KRAS‐selective inhibitors such as AMG510 and MRTX849 targeting KRAS‐G12C [18]. In our previous research, we reported that the “open conformation” on the surface of HRASG60A‐GppNp might be a potential target for KRAS‐driven non‐small cell lung cancer therapy [19]. In *in silico* docking models (Figure 2A), NSC48160 (named as 4‐tert‐butyl‐2‐[(cyclohexylamino) methyl]‐6‐methylphenol) was trapped in the binding cavity between the switch 1 region and the relevant residues (GLN25, ILE‐21, ASP‐30, TYR‐32). SPR analysis was conducted using the recombinant protein of RAS‐G60A‐GppNp for further confirmation, and the binding curves of association/dissociation and the steady‐state analysis disclosed a high affinity with a KD value of 19.2 μM (Figure 2B). Then, a CETSA experiment was performed in HepG2 and SMMC‐7721 cells to verify the in vivo interaction between NSC48160 and the KRAS protein. The thermal stability of KRAS was enhanced to varying degrees with the addition of NSC48160 (36 μM) ranging from 47°C to 63°C (Figure 2C), indicating possible interactions between NSC48160 and KRAS in vivo. Incorporating the pharmacological agent significantly modified the conformation of KRAS proteins, which in turn influenced the status of tumor cells. For instance, upon the introduction of NSC48160 (20–40 μM), a notable reduction (red line) in the cellular confluence of HCC cells was observed (Figure 2D–F). In accordance with this, NSC48160 significantly inhibited cell proliferation in these cells (Supporting Information S1: Figure S2A) and inhibited colony formation (Supporting Information S1: Figure S2B). This reduction was directly proportional to both the concentration of the administered drug and the duration of exposure. Remarkably, normal hepatocytes exhibited robust resistance to these changes, remaining largely unaffected under similar conditions (Figure 2G). In conclusion, NSC48160 exhibited promising potential as a therapeutic agent in HCC.

### 3.3 NSC48160 Alleviates Tumor Progression by Reprogramming the TCA Cycle Metabolism and Promoting the Ferroptosis

**FIGURE 3:**
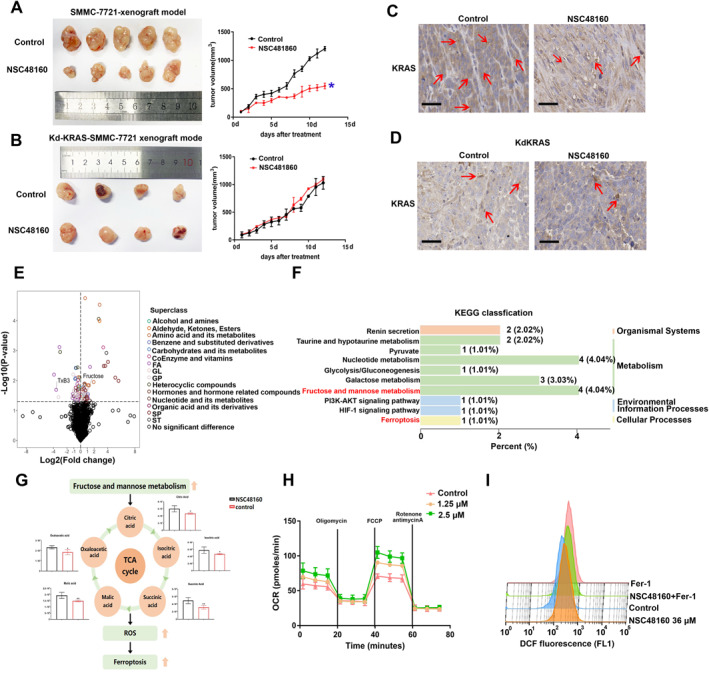
NSC48160 shows an evident effect of tumor growth inhibition and metabolic changes. (A and B). The representative images of burdened tumors in the control, NSC48160 treatment group of SMMC‐7721 xenograft model and KRAS‐knockdown‐SMMC‐7721 xenograft model. And the tumor growth curve each group is on the right. (C and D) The immunohistochemical analyses of KRAS were examined in the indicated groups. Scale: 50 μm. *n* = 3. (E) Volcano plots of differential metabolites in the control (*n* = 6) and NSC48160 (*n* = 6) groups. Tumor tissues from the indicated were used for the analysis. (F) KEGG pathway enrichment results show glycolysis, fructose metabolism, and ferroptosis‐related pathways. (G) The peak area of intermediate metabolites of the TCA cycle. *n* = 6 per group. (H) Mitochondrial respiration and glycolysis of SMMC‐7721 cells together with NSC48160 (1.25 μM, 2.5 μM). (I) 3D plot showing ROS production. Tumor cells were treated with NSC48160, ferroptosis inhibitor Fer‐1, and a combination of the two ingredients. Data represent the mean ± SEM. *n* = 3, *p < 0.05, **p < 0.01.

To investigate the relationship between metabolic response and HCC progression, we first set up an HCC xenograft mouse model. With a stable genetic background, SMMC‐7721 and SMMC‐7721‐knockdown KRAS cells were respectively injected s.c. into the flanks of 5‐6‐week‐old Balb/c nude mice treated with vehicle chow or NSC48160 through daily intraperitoneal injection after tumor genesis. As shown in Figure 3A, continuous NSC41860 treatment led to a prolonged tumor growth inhibition which can be shown by tumor growth curves. The tumor tissue dimensions of the knockdown‐KRAS‐SMMC‐7721 xenograft model have no changes after treatment of NSC48160. The knockdown‐KRAS‐SMMC‐7721 xenograft model also showed no significant changes in tumor weight and tumor volume (Supporting Information S1: Figure S3A B). NSC48160 also showed no toxicological response (Supporting Information S1: Figure S3C). IHC analysis showed that KRAS expression was lower in the NSC48160 treatment group compared with the control group (Figure 3B). Similarly, subcutaneous injections of cells knocked down for KRAS into tumors was found to have no significant effect on treatment with NSC48160 (Figure 3C, Supporting Information S1: Figure S3). Consistent with the above findings, the subgroup of subcutaneous implant tumors derived from SMMC‐7721 cells with KRAS‐knockdown exhibited negligible changes in the level of KRAS expression treatment with NSC48160 (Figure 3D). Recent insights indicate that ferroptosis is intrinsically linked with multiple cellular metabolic pathways, including energy and carbohydrate metabolism. To explore how the metabolic microenvironment affects HCC progression, we performed metabolic profiling of tumor tissues. Significant accumulation of metabolites (Fructose and TxB3) related to the TCA cycle, carbohydrate metabolism, and ferroptosis were found in NSC48160 treatment group mice (Figure 3E,F). The occurrence of fructose and mannose metabolism disorder was often associated with cancer. NSC48160 increased fructose metabolism and augmented the TCA cycle, which can be proved by intermediate metabolites of the TCA cycle (Citric acid, Malic acid, Isocitric acid, Succinic acid, and Oxaloacetic acid) (Figure 3G). Meanwhile, the mitochondrial OXPHOS activity in the NSC48160‐treated tumor cells has been evidently augmented (Figure 3H). We found that the ferroptosis inhibitor Fer‐1 reduced ROS production. However, the addition of NSC48160 promoted ferroptosis and ROS production simultaneously (Figure 3I). The metabolic reprogramming after NSC48160 treatment would elevate the cellular ROS levels to disrupt iron homeostasis and promote ferroptosis.

### 3.4 NSC48160 Exhibits Sensitivity to Ferroptosis in Hepatocellular Carcinoma

**FIGURE 4:**
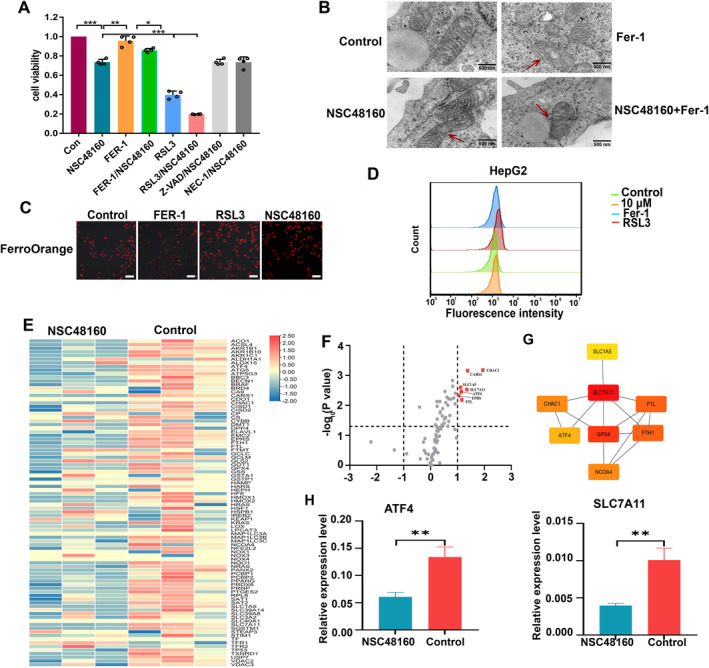
NSC48160 increases sensitivity to ferroptosis. (A) Effect of NSC48160 (36 μM) on cell death. HepG2 cells were treated with NSC48160 alone or in combination with Fer‐1, RSL3, NEC‐1, or Z‐VAD for 24 h, and cell viability was measured. Survival data for the NSC48160 group and the above group were normalized to the untreated control group, the Fer‐1‐alone, and the RSL3‐alone group, respectively. (B) Mitochondria morphology. Red arrowheads indicate the creation of mitochondria. Scale bar: 500 nm. (C) Ferro‐orange staining for intracellular Fe^2+^ in HepG2 cells treated for 24 h in the presence of RSL3 (1 μM), FER‐1 (1 μM), or NSC48160 (36 μM). Shown is one of three representative fields illustrating fluorescence intensity taken at identical exposures for each condition. Scale bar, 20 μm. (D) Effects of NSC48160, Fer‐1, and RSL3 on ROS production in HepG2 cells. (E) Heatmap showing significantly differently expressed metabolites (*p* < 0.05) between control and treatment groups. Values are scaled as indicated (2 to −2) (*n* = 3). (F) The volcano map shows significantly different genes, SLC7A11, ATF4, FTL, etc. (G) Protein‐protein interaction (PPI) network of ferroptosis from metabonomics. (H) Expression of genes related to ferroptosis in hepatocytes from the indicated groups, *n* = 3.

To examine whether NSC48160 increased susceptibility to ferroptosis, hepatocytes (HepG2 cells) were treated with different cell death inducers or inhibitors, including the ferroptosis inducer RAS‐selective lethal 3 (RSL3), ferroptosis inhibitor ferrostatin‐1 (Fer‐1), caspase inhibitor Z‐VAD, and necroptosis inhibitor necrostatin‐1 (NEC‐1). Fer‐1 completely inhibited hepatocyte death, while Z‐VAD or NEC‐1 was ineffective (Figure 4A). The cells died after incubation with low RSL3 concentrations, when NSC48160 was added, hepatocytes exhibited more cell death, indicating a greater susceptibility to ferroptosis. Further, the transmission electron microscopy assay revealed that cells subjected to NSC48160 treatment demonstrated mitochondrial crenation. In contrast, the application of Fer‐1 did not result in any noticeable morphological alterations in HepG2 cells (Figure 4B). Since ferroptosis was mainly characterized by intracellular iron overload and redox imbalance, we first measured the level of intracellular iron content by using FerroOrange. Similar to the ferroptosis inducer (RSL3), NSC48160 could ignite excessive iron content in HepG2 cells, while these behaviors can be remarkably alleviated by ferroptosis inhibitor Fer‐1 to some extent (Figure 4C). We performed flow cytometry to measure ROS levels. As shown in Figure 4D, Treatment with the ferroptosis inducer (RSL3) and inhibitor (Fer‐1) markedly increased or reduced the ROS levels, respectively. We supposed that NSC48160 increased total ROS levels in HepG2 cells by promoting ferroptosis. To further understand the mechanism of NSC48160 on ferroptosis, we performed a ferroptosis PCR array on control and NSC48160‐treated cells to analyze the gene expression pattern in the ferroptosis‐related signaling pathway. Among genes with significant fold‐change, ferroptosis suppressers SLC7A11 and ATF4 were the most downregulated (Figure 4E,F). According to the CytoHubba plug‐in MCC algorithm in Cytoscape software, the top two genes (SLC7A11 and GPX4) with high core degrees were the hub genes (Figure 4G). And the sub‐networks from complex interactome consisted of ferroptosis‐related genes, including ATF4 and FTL. Cellular assays also confirmed that SLC7A11 and ATF4 expression levels were reduced in the NSC48160 treatment group (Figure 4H). In theory, NSC48160 inhibited the transcription factor ATF4, resulting in inhibiting the transcription of SLC7A11, and then promoted ferroptosis.

### 3.5 Synergetic Inhibition of SLC7A11 Promotes NSC48160‐Inducing Ferroptosis

**FIGURE 5:**
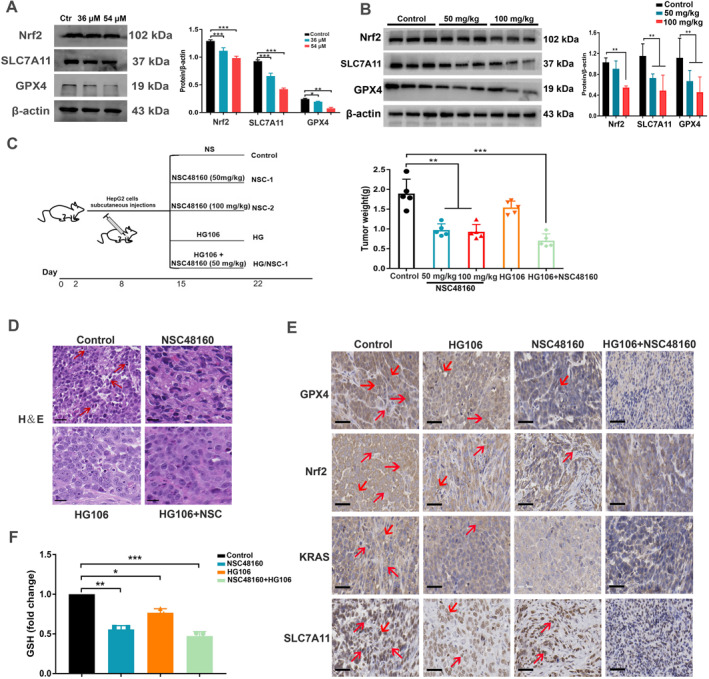
The synergy between NSC48160 and HG106 successfully reversed tumor suppression in vivo. (A) NSC48160 inhibited the Nrf2/SLC7A11/GPX4 signal pathway. HepG2 cells were treated with NSC48160 (36 μM/54 μM) for 24 h. (B) SLC7A11, Nrf2, GPX4 expression in tumors from Balb/c nude mice (*n* = 3). (C) Schematic illustration of the animal test and drug treatment protocol. After a 7‐day tumor formation, mice were treated with vehicle NSC48160 (50 mg/kg, 100 mg/kg) or HG106 (1 mg/kg) and a combination of NSC48160 (50 mg/kg) and HG106 (1 mg/kg) for an additional 7 days. And the histogram (right) shows the tumor weights at the endpoint of the indicated group (*n* = 5). The vertical bars represent the range of the data. All data are shown as mean ± SD. **p < 0.01; ***p < 0.001. (D) Representative H&E staining of liver sections of mice (*n* = 5). Scale bars: 50 μm. (E) SLC7A11, Nrf2, GPX4 expression in tumors from the indicated group (*n* = 3). Scale bars: 50 μm. (F) The relative GSH levels were measured in indicated mice tumor tissues (control group, NSC48160 group with or without HG106. Data are presented as mean ± SD. *p < 0.05; **p < 0.01; ***p < 0.001.

SLC7A11 is a key regulator of ferroptosis, which is over‐expressed in HCC [20, 21]. To investigate whether KRAS/SLC7A11 was the primary factor in ferroptosis, the expression of SLC7A11 in KRAS‐mutant HCC cells was tested experimentally. Obviously, NSC48160 downregulated the SLC7A11 protein level in SMMC‐7721 cells (Figure 5A). In contrast, the protein level of Nrf2, a specific transcription factor of SLC7A11, showed high expression in SMMC‐7721 cells (Figure 5A), while NSC48160 decreased the expression of Nrf2. GPX4, a key ferroptosis index targeting the SLC7A11‐GPX4 regulatory axis responsible for inducing ferroptosis, was significantly suppressed by NSC48160 treatment (Figure 5A). Aiming to verify the findings, SLC7A11 protein in mouse liver tumors was examined further. As displayed in Figure 5B, WB analyses revealed a significant decrease in SLC7A11 expression in NSC48160‐treated tumor tissues in a dose‐dependent manner. Similar results were also observed in GPX4 and Nrf2 protein levels (Figure 5B). To identify the central role of SLC7A11, pharmacological inhibitor HG106 was further used to specifically block SLC7A11 expression in SMMC‐7721‐based xenograft mice (Figure 5C). Results suggest that NSC48160 and HG106 inhibit tumor growth commonly. It was observed that the group treated with pharmaceutical intervention exhibited no discernible pathological alterations in tumor tissues when compared to the control group, as evidenced by H&E staining (Figure 5D). The inhibitory effect of the SLC7A11 antagonist, referred to as HG106, was observed to promote ferroptosis, through the degradation of GPX4 (Figure 5E). Concurrently, the group treated with NSC48160 exhibited a marked suppression of the Nrf2‐SLC7A11‐GPX4 signaling pathway alongside a decrease in KRAS expression, further facilitating ferroptosis. Importantly, ferroptosis induction was observed to be significantly more pronounced in the synergy of NSC48160 and HG106 (Figure 5E), implying a cooperative regulatory mechanism. Given that ferroptosis is caused by the loss of cell redox balance, glutathione (GSH) played a vital role in eliminating the accumulation of lipid ROS. We next investigated whether NSC48160 influenced GSH synthesis and quantified the levels of GSH with or without HG106 treatment (Figure 5F). Taken together, NSC48160 could induce ferroptosis by targeting the KRAS‐SLC7A11‐GPX4 regulatory axis to inhibit the synthesis of GSH.

### 3.6 SLC7A11 Is Key for NSC48160 to Regulate Metabolic Reprogramming

**FIGURE 6:**
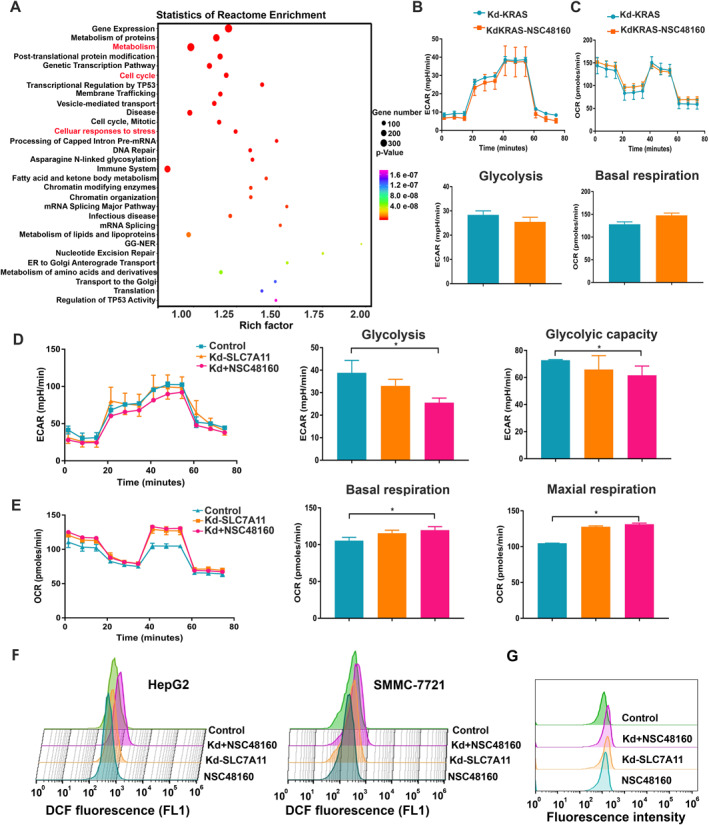
NSC48160‐SLC7A11 knockdown combination remodels metabolic phenotype. (A) The top 20 pathways in rich factors of gene pathway enrichment. The rich factor indicates the ratio of the number of differentially enriched genes to the number of annotated genes in this pathway, and the number indicates the number of genes enriched in this pathway. (B and C) Effect of knockdown KRAS on the real‐time mitochondrial respiration and the real‐time glycolysis with or without NSC48160. HepG2 cells treated with NSC48160 (1.25 μM) for 24 h were evaluated for mitochondrial OCR and the extracellular acidification rate in Seahorse testing. (D and E) The effect of NSC48160 synergy with knockdown SLC7A11 on the Seahorse metabolic analysis was performed to examine the OCR and ECAR in HepG2 cells. Quantitative data are expressed as mean ± SD. *p < 0.05, as compared with the control. (F) Flow cytometric analysis of intracellular ROS in HepG2 and SMMC‐7721 cells treated with DMSO, NSC48160 with or without knockdown SLC7A11. (G) The lipid peroxidation detection in the above group. (*n* = 3, *p < 0.05).

Mitochondria play an indispensable role in regulating energy metabolism [22]. SLC7A11 takes up a high level of cystine from the extracellular environment to generate more GSH [23], sustaining the oxidative balance in HCC cells. The function of the genes identified as upregulated in the NSC48160 group in vivo was pursued using KEGG pathway analysis (Figure 6A). The pathways upregulated by NSC48160 specifically were linked predominantly to metabolism, cell cycle, and cellular response to stress. Since the knock‐down KRAS have the capacity to inhibit cellular homeostatic survival in cancer cells, the knock‐down KRAS HCC cell (SMMC‐7721) also exhibits low glycolytic metabolism (Figure 6B). Upon adding NSC48160 to cells with knock‐down KRAS, the oxygen consumption rate (OCR) data showed that both the basal respiration rates remained largely unaltered (Figure 6C). Collectively, reprogramming cellular metabolism caters to the elevated energy and biomass requirements necessitated by uncontrolled proliferation. Furthermore, diminishing the expression of KRAS impedes anabolic pathways, thereby mitigating the metabolic alterations characteristically associated with cancer. Based on previous research, inhibiting SLC7A11 activities could lead to ferroptosis in HCC cells [24]. To observe the effect of SLC7A11 on energy regulation homeostasis, we proceeded to knock down SLC7A11 within SMMC‐7721 cells. With the addition of NSC48160, results showed a decreased glycolysis compared to both the control and knock‐down SLC7A11 cells (Figure 6D), but increased mitochondrial respiration becomes the primary energy source in the knock‐down SLC7A11 SMMC‐7721 cells (Figure 6E). This trend means that SLC7A11 is key for NSC48160‐mediated metabolic reprogramming. An excessive enhancement in mitochondrial respiratory capacity resulted in the overproduction of ROS, thereby leading to functional damage of mitochondria. The reduced membrane potential caused by the addition of NSC48160 further supported the relationship between mitochondrial respiratory and ROS production in both the HepG2 and SMMC‐7721 cell lines (Figure 6F). ROS generation is also promoted by excessive iron and then accelerates lipid peroxidation. NSC48160 improved lipid peroxidation, and the effects are more evident in the knockdown of SLC7A11 with the NSC48160 group in HepG2 cell lines (Figure 6G). In conclusion, the addition of NSC48160 not only modulated the metabolic phenotype but also balanced the oxidative stress in the HCC cells knocked down SLC7A11.

## 4 Discussion

The RAS proteins are essential molecular switches responsible for receiving signals from various external sources and transmitting them to various internal effectors [25, 26]. Previous research has found that KRAS is upregulated in a variety of human cancers and that this upregulation contributes to tumorigenesis [27, 28]. HCC is a type of cancer that is typically caused by hepatitis B or C virus infection, alcohol abuse, and various metabolic disorders [29, 30, 31]. Our research indicates that KRAS is significantly increased in HCC and linked to poor prognosis for HCC patients (Figure 1). It would be noteworthy to investigate which metabolic risk factors contribute to the disease progression. Moreover, given the high levels of KRAS expression in cancer, there is considerable interest in developing therapies that target HCC with KRAS expression.

The introduction of AMG 510 and MRTX849, which are designed to target KRAS (OFF) status, represents a significant advancement in the field of oncology. However, the clinical application of these agents is hampered by multifaceted and intricate resistance mechanisms. This synergistic approach has yielded promising outcomes in suppressing tumor growth across a diverse array of cancer models [32]. To investigate the effects of multi‐targeted therapies, we conduct a thorough analysis of metabolic dysregulation in HCC cells targeting KRAS with NSC48160 to identify metabolic vulnerabilities that could be targeted in treating HCC. Our findings reveal significant metabolite changes upon KRAS inhibition, particularly in intermediates in the TCA cycle and fructose metabolism (Figure 3). Aberrations in the antioxidant system and the TCA cycle, driven by the catabolism of glutamine, precipitate disturbed mitochondrial respiration, thereby facilitating the overproduction of ROS. This phenotypic alteration significantly impacts metabolic processes within the cell. The metabolic framework of tumor cells is pivotal in regulating mitochondrial impairment, coupled with elevated levels of oxidative stress and lipid oxidation; thus, perturbations in the metabolic operations of these cells render them increasingly susceptible to ferroptosis (Figure 4). The significance of cellular energy metabolism and ion metabolism in the context of ferroptosis in tumor cells is paramount. Cellular energy metabolism, encompassing glucose, fatty acid, and amino acid metabolism, not only serves to furnish the necessary energy for cellular proliferation but also plays a critical role in the regulation of intracellular redox homeostasis [33]. This regulation is mediated through the modulation of intracellular concentrations of NADH, NADPH, GSH, and ROS, all of which can influence the behaviors of ferroptosis in neoplastic cells. Furthermore, ion metabolism, specifically concerning iron and selenium ions, has a profound impact on enzymatic activity and subsequently modulates cellular ferroptosis. This is achieved through the ions' direct involvement in lipid peroxidation as well as their function as essential cofactors in the synthesis of enzymes that are intricately associated with the ferroptosis pathway [34].

As the most critical regulator of ferroptosis in HCC, SLC7A11 centrally regulates adaptive glutathione metabolism by conferring specificity for cystine uptake, which was found to be highly overexpressed in HCC cells and tissues and displayed a significant association with tumor progression. According to our research, targeting SLC7A11 significantly impaired the growth and survival of HCC in vitro and in vivo, indicating that it is a viable functional target. Our study integrated NSC48160 with the SLC7A11 inhibitor HG106 as a therapeutic intervention for HCC, indicating that this combination yields a significant inhibitory effect (Figure 5). This heightened sensitivity augments the efficacy of tumor cell eradication. The strategic integration of ferroptosis inducers with KRAS inhibitors may herald a novel paradigm in cancer therapeutics [35, 36].

**FIGURE 7:**
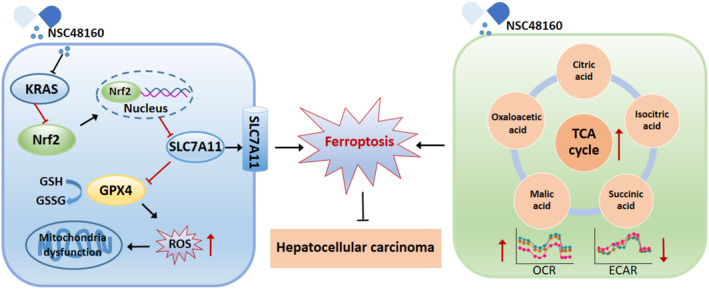
Molecular target engagements and metabolic changes reveal that NSC48160 inhibits the progression of HCC through ferroptosis. NSC48160 elucidates the ferroptosis mechanism whereby it impedes KRAS and instigates the downregulation of Nrf2/SLC7A11/GPX4. Meanwhile, NSC48160 inhibits HCC by promoting the TCA cycle, elevating the OCR, and an augmented generation of ROS.

Our study identifies NSC48160 as a targeting agent that disrupts KRAS‐driven metabolic adaptation and ferroptosis resistance. By enhancing TCA cycle activity, NSC48160 elevates mitochondrial ROS production, overwhelming the Nrf2‐SLC7A11‐GPX4 axis and triggering lipid peroxidation (Figure 6). This mechanism aligns with recent evidence that KRAS‐mutant cancers exhibit heightened dependence on redox homeostasis (Figure 7). Notably, combining NSC48160 with the SLC7A11 inhibitor HG106 achieved synergistic tumor suppression, suggesting a paradigm for co‐targeting oncogenic signaling and ferroptosis vulnerability.

## Author Contributions

**Jiaxin Zhang:** conceptualization, methodology, investigation, writing – original draft, resources. **Zuojia Liu:** conceptualization, funding acquisition, resources, supervision. **Wenjing Zhao:** investigation. **Chang Li:** methodology. **Fei Liu:** investigation. **Jin Wang:** conceptualization, resources, supervision.

## Ethics Statement

Cancer patients enrolled in this study were those pathologically diagnosed with liver cancer. The written informed consents were obtained from all participants before tissue collection for research according to regular principles. The Ethics Committees of the Hepatobiliary Hospital of Jilin Province approved all human tissue protocols. The animal study was reviewed and approved by the Animal Care and Use Committee of the College of Basic Medical Sciences of Jilin University, Changchun, China (Approval Number: 2024‐17, Changchun, China).

## Conflicts of Interest

The authors declare no conflicts of interest.

## Supporting information

Supporting Information S1
