# Supplementary material for: Targeting KRAS Sensitizes Ferroptosis by Coordinately Regulating the TCA Cycle and Nrf2‐SLC7A11‐GPX4 Signaling in Hepatocellular Carcinoma
Source: Smart Med. 2025 May 6;4(2):e70005. doi: 10.1002/smmd.70005 (PMC12087391; doi:10.1002/smmd.70005)
Supplement: Supplementary file 1 — Supporting Information S1 [file SMMD-4-e70005-s001.docx]

**Targeting KRAS sensitizes ferroptosis by coordinately regulating the TCA cycle and Nrf2-SLC7A11-GPX4 signaling in hepatocellular carcinoma**

Jiaxin Zhang^a,b^, Zuojia Liu^a,*^, Wenjing Zhao^c^, Chang Li^a^, Fei Liu^d^, Jin Wang^d, e *^

*^a^* State Key Laboratory of Electroanalytical Chemistry, Changchun Institute of Applied Chemistry, Chinese Academy of Sciences, Changchun 130022, China

*^b^* School of Chinese Medicine, Hong Kong Traditional Chinese Medicine Phenome Research Center, Hong Kong Baptist University, Hong Kong 999077, China

*^c^* Hepatobiliary Hospital of Jilin Province, Changchun 130062, China

*^d^* Center for Theoretical Interdisciplinary Sciences, Wenzhou Institute, University of Chinese Academy of Sciences, Wenzhou, Zhejiang 325001, China

*^e^* Department of Chemistry and Physics, Stony Brook University, Stony Brook, New York 11794-3400, USA

^*^Corresponding authors. State Key Laboratory of Electroanalytical Chemistry, Changchun Institute of Applied Chemistry, Chinese Academy of Sciences, No. 5625 Renmin Street, Changchun 130022, Jilin, China. Email address: zjliu@ciac.ac.cn (Z. Liu).

Center for Theoretical Interdisciplinary Sciences, Wenzhou Institute, University of Chinese Academy of Sciences, No.1 Jinlian Road, Wenzhou 325001, Zhejiang, China. Email address: jin.wang.1@stonybrook.edu (J. Wang).


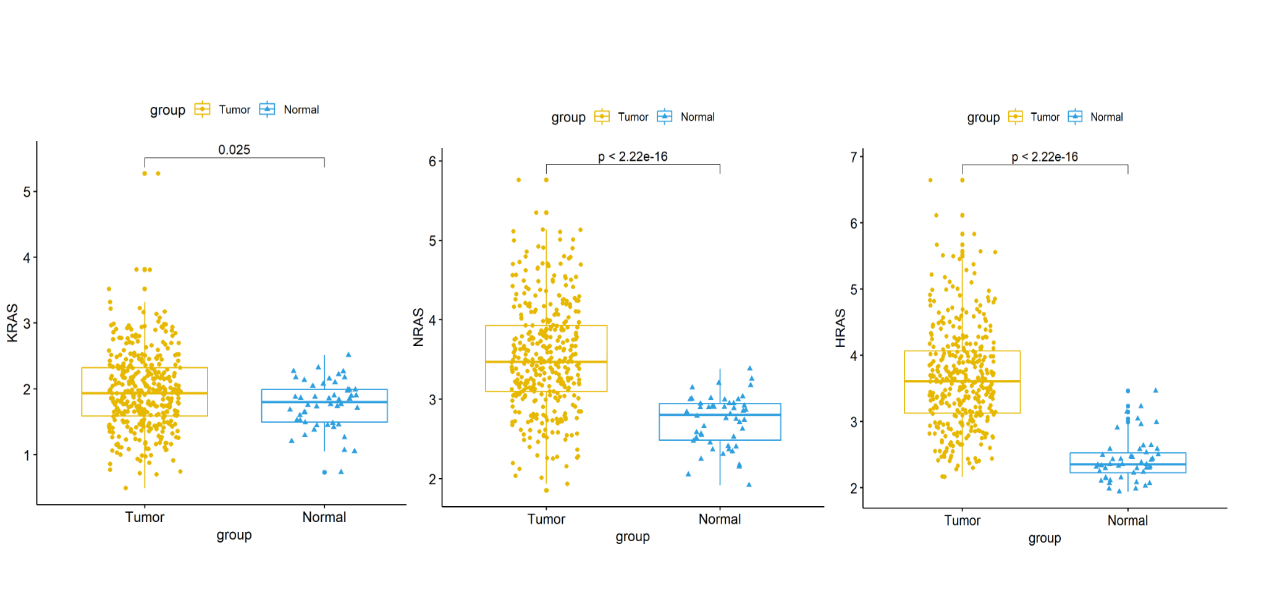


Figure S1. The expression of KRAS, HRAS, and NRAS in tumor tissues and normal tissues.


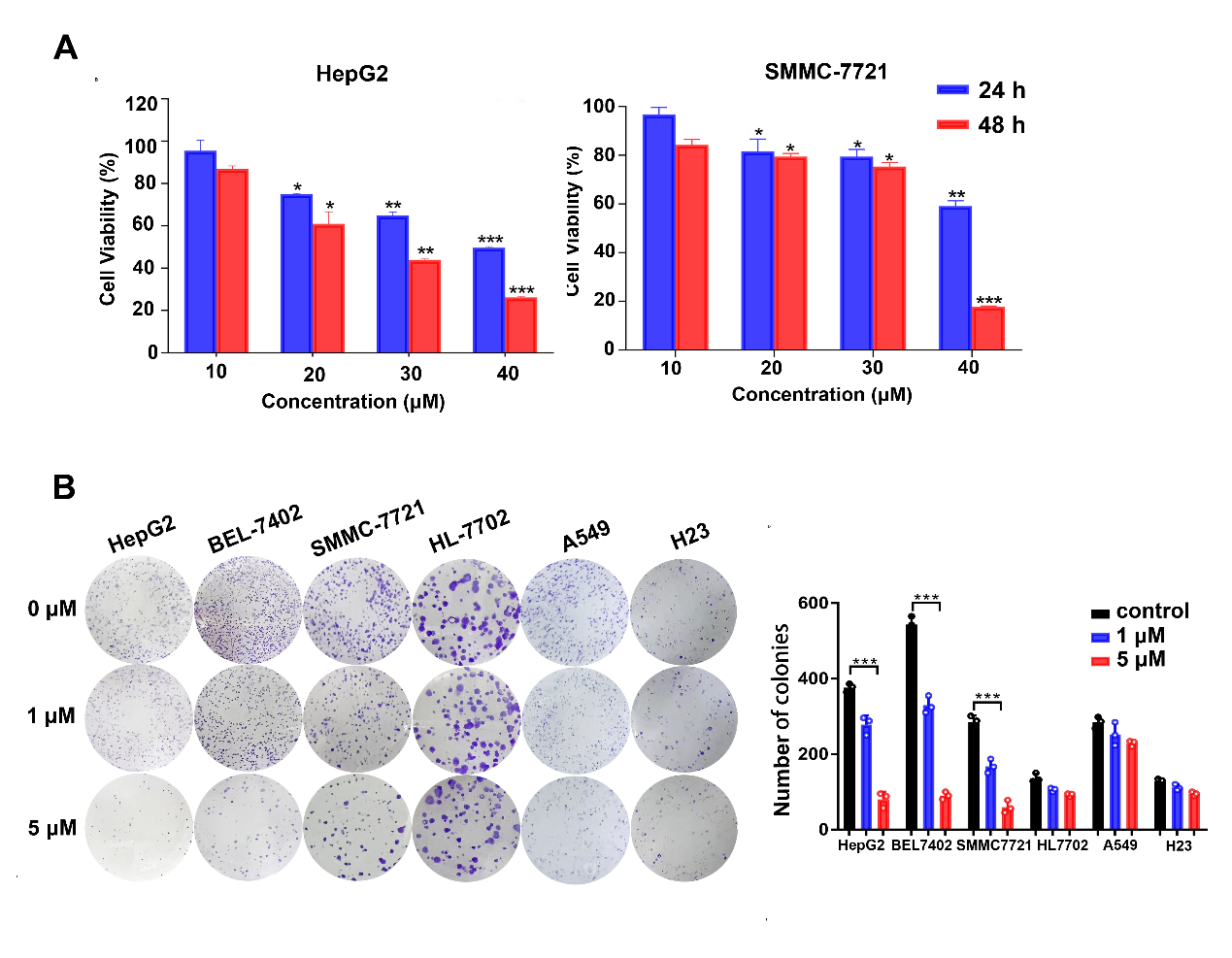


Figure S2. (A) Effect of NSC48160 (10, 20, 30, 40 μM) on cell death. HepG2 and SMMC-7721 cells were treated with NSC48160 alone for 24 and 48 hours, and cell viability was measured. (B) The effect of NSC48160 on HepG2, BEL-7402, SMMC-7721, HL7402, A549, H23 cell colony formation. The colony number was normalized to the control.


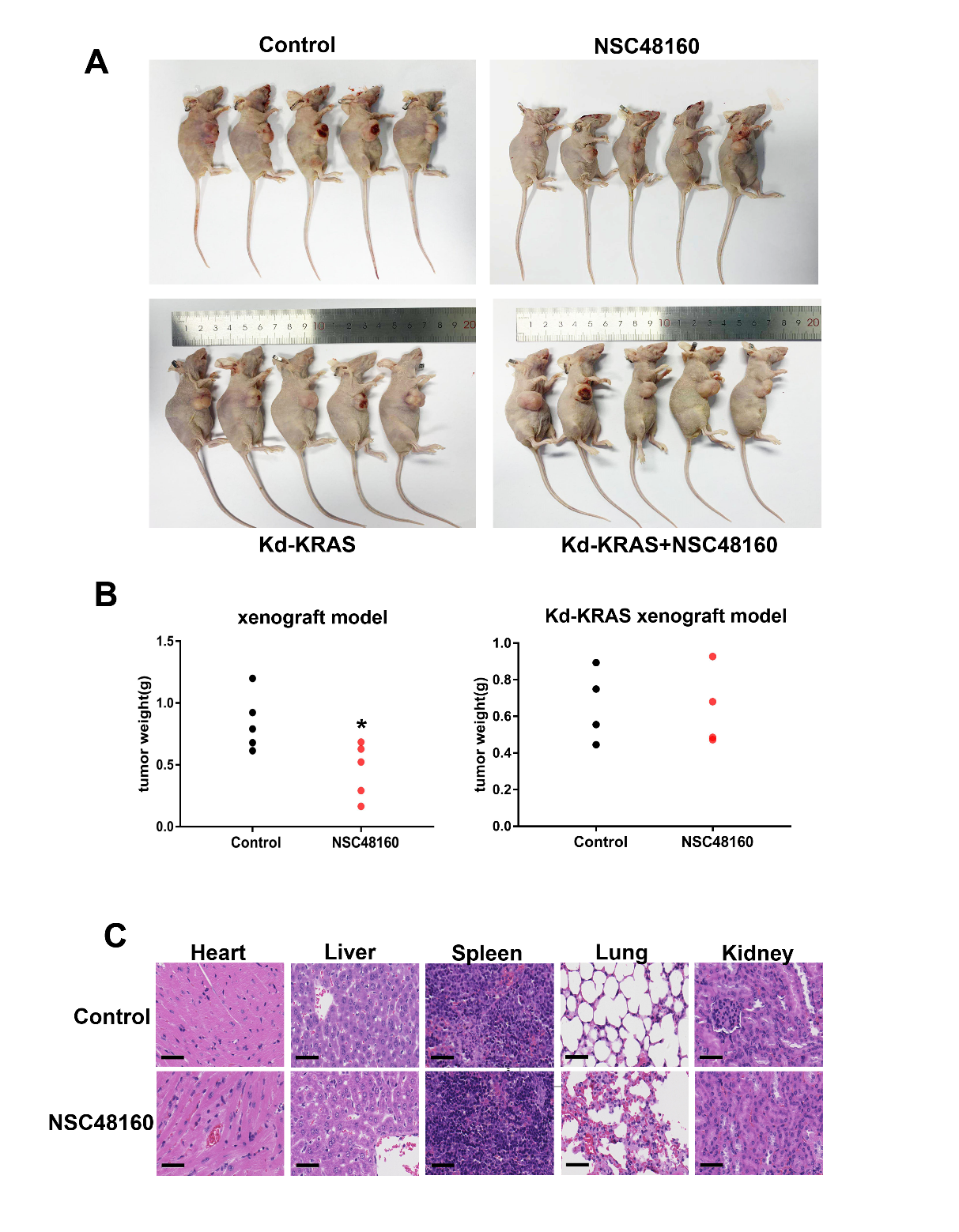
Figure S3. (A) The representative images of burdened tumors in each group. (B)Tumor volume weight of SMMC-7721 xenograft model and knockdown-KRAS-SMMC-7721 xenograft model. (C) H&E staining for the lung tissue in each group. Scale: 50 μm.
